# Exploring Opportunities and Challenges for the Spread, Scale-Up, and Sustainability of mHealth Apps for Self-Management of Patients With Type 2 Diabetes Mellitus in the Netherlands: Citizen Science Approach

**DOI:** 10.2196/56917

**Published:** 2024-12-17

**Authors:** Catharina Margaretha van Leersum, Marloes Bults, Egbert Siebrand, Theodorus Johannes Josef Olthuis, Robin Enya Marije Bekhuis, Annemieke Ariënne Johanneke Konijnendijk, Marjolein Elisabeth Maria den Ouden

**Affiliations:** 1 Department of Technology, Policy, and Society Faculty of Behavioural, Management, and Social Sciences University of Twente Enschede Netherlands; 2 Faculty of Humanities Open Universiteit Heerlen Netherlands; 3 Technology, Health & Care Research Group Saxion University of Applied Sciences Enschede Netherlands; 4 Ethics and Technology Research Group Saxion University of Applied Sciences Deventer Netherlands; 5 Care & Technology Research Group Regional Community College of Twente Hengelo Netherlands; 6 Department of Smartup Innovation Ziekenhuisgroep Twente Almelo Netherlands; 7 Santeon Utrecht Netherlands

**Keywords:** mHealth, type 2 diabetes mellitus, implementation, self-management, health care system, citizen science, mobile health, mobile app, digital health, digital technology, digital intervention, smartphone, diabetes, DM, type 2 diabetes, type 1 diabetes

## Abstract

**Background:**

Technologies evolve at a breakneck pace, and the success of mobile health (mHealth) for people with type 2 diabetes mellitus (T2DM) depends on whether health care professionals, care management, government regulators, and consumers will adopt the technology as a viable solution to enhance patient self-management.

**Objective:**

In this study, we explored the challenges of the implementation of mHealth apps in care for patients with T2DM and determined to what extent these challenges complicate the dissemination, limit scale-up, and influence the sustainability of technological interventions for patients with T2DM.

**Methods:**

The nonadoption, abandonment, and challenges to scale-up, spread, and sustainability (NASSS) framework served as the basis for our study. The 7 domains of the NASSS framework were explored with a citizen science approach using questionnaires, semistructured in-depth interviews, and focus groups together with patients with T2DM, care professionals, technology developers, policy officers, and a patient organization.

**Results:**

Regarding the domain “condition,” being aware of their condition and changing lifestyle were crucial for patients with T2DM to get to grips with their life. The rapid development of health apps for T2DM was highlighted in the domain “technology.” Users should be aware of these apps and know how to use them. The domain “value proposition” included the patient perspective and elaborated on personal values, as well as care professionals who focus on personalized care and pressure on health care. Regarding the “adopters,” it is crucial to know who needs to use and introduce the apps. Responsibility, a shared vision, and resistance among care professionals were mentioned as important determinants for “organization.” Finally, the domain “wider system” showed the importance of involving multiple institutes, care guidelines, and reimbursements.

**Conclusions:**

This study investigated the implementation of mHealth apps in an early stage of the implementation process. Key stakeholders were involved, who attributed to the possibilities and limitations of the implementation. It is crucial to have a clear vision from an organizational perspective and specific prerequisites for implementation strategies at micro, meso, and macro levels. Essential strategies at the national level include guidelines for regulations, privacy, and security; the integration of mHealth into T2DM care guidelines; and sufficient reimbursement by health insurers.

## Introduction

Health technologies evolve at a breakneck pace. The future success of mobile health (mHealth) for patients with type 2 diabetes mellitus (T2DM) depends on whether health care professionals, care management, government regulators, and patients will adopt the technology to enhance patient self-management [1]. During past years, a large number of mHealth apps for T2DM have been developed and evaluated, for example, to improve self-management, provide lifestyle coaching, or provide continuous glucose monitoring. These apps can have a positive impact on patients with T2DM, that is, improving hemoglobin A_1c_ levels, medication adherence, and facilitating self-management [2,3]. Although there is a wide array of evidence-based mHealth apps for patients with T2DM available, apps often stand alone and are not integrated into care pathways. Care professionals mainly experience time and work pressure–related barriers, but their familiarity with apps is low as well [2]. Hence, the development of strategies to support the incorporation of mHealth apps in care practices is needed. These strategies can include the integration of advice on the use of apps in existing care pathways to improve knowledge regarding self-management of diabetes for the patients and assist professionals to provide personalized care. In Norway, mobile apps are recommended within diabetes guidelines to track physical activity in combination with blood glucose monitoring [4]. In the United States, guidelines take a stance toward preventing the development of T2DM with the use of mHealth [5]. However, the guidelines of the European Association for the Study of Diabetes do not recommend the use of technology [6].

In previous research, we applied so-called citizen science research methods to explore the acceptability, experiences, and acceptance of the use of mHealth by patients with T2DM [7]. Most patients with T2DM were positive about the use and potential added value of mHealth apps [7]. However, both patients and professionals experienced difficulties in incorporating the collected personal data into care pathways and consultations [8,9]. Also, in other studies, it was found that mHealth was not structurally implemented as integral part of care pathways [10]. There is a need to learn more about approaches that can promote the integration of mHealth in the care of patients with T2DM among a variety of health care stakeholders, including care professionals, technology developers, policy officers, and patient organization.

In this study, we aimed to explore the challenges of the implementation of mHealth apps in care pathways for patients with T2DM and determine to what extent these challenges complicate the dissemination, limit scale-up, and influence sustainability of technological interventions for T2DM. The nonadoption, abandonment, and challenges to scale-up, spread, and sustainability (NASSS) framework served as the basis for this study [11]. This framework is useful to incorporate the perspectives of different stakeholders in different research and development stages of technological innovations in health and social care [11]. The framework consists of seven domains: (1) condition, (2) technology, (3) value proposition, (4) adopters, (5) organization, (6) wider system, and (7) embedding and adaptation over time. The different domains were used to provide insight into the challenges that stakeholders experience when using and implementing technology.

## Methods

### Design and Setting

This research is based on data from previous research [7-9,12] and newly obtained data. Although all data were used to take perspectives of different stakeholders into consideration and analyze all the domains of the NASSS framework, not all datasets contributed equally to each domain. In our study design, we followed the NASSS toolkit of Greenhalgh et al [13]. They described which stakeholders seemed the best fit to complete each domain. In this toolkit, the stakeholders that should be part of each domain: (1) domain 1 should include the clinicians, social workers, or researchers; (2) domain 2 should include the technology developers; (3) domain 3 should include the technology developers and business lead for the organization; (4) domain 4 should include stakeholders on behalf of everyone who might use the technology; (5) domain 5 should be discussed by people who know the organization and the challenges it faces, for example, board member, human resources lead, and staff representative; (6) domain 6 should include a “horizon-scanner” who looks beyond the organization; and (7) domain 7 should pull together the bottom row of each of the previous domains. All data collection steps and thereby all stakeholders contributed to each domain. However, some datasets acquired from specific stakeholders contributed more to a certain domain and less to the other domains.

We were able to use the previously acquired data mostly to discuss the first, second, third, and fourth domains. However, the data can as well partly contribute to the analysis of the other domains. The previous data collection included questionnaires among patients with T2DM on their desired involvement in citizen science research [12], questionnaires with follow-up interviews considering the use of technology by patients with T2DM [8], and interviews and follow-up focus groups with patients and lifestyle coaches about their expectations and experiences before and after testing mHealth apps for patients with T2DM [7]. We obtained new data to mainly discuss the third, fourth, fifth, sixth, and seventh domains of the NASSS framework, but the new data also added relevant information to the first and second domains. The cross-pollination of previous and newly collected data led to the incorporation of perspectives of different stakeholders in each of the domains. To obtain new data, we applied a qualitative research design with a citizen science approach [14] to explore the subjective expectations, perceptions, and experiences of different stakeholders involved in the care of patients with T2DM in the Netherlands.

This study was part of TOPFIT Citizenlab, a 3-year research and innovation program in the Netherlands. Here, citizens, care professionals, and companies joined forces with researchers to develop and implement technology for health and well-being. The stakeholders involved were not only respondents of the research but became coresearchers who had an active role in the design, data collection, or analysis process.

### Recruitment

To involve different stakeholders in this study, we used a variety of recruitment strategies. First, the 4 involved development companies in our previous study [8] were asked to continue collaboration. Second, flyers and announcements on social and regional media were used to recruit health care professionals. Third, purposive sampling was used to include a specific group of stakeholders. We reached out to project managers, insurance companies, policy officers of care organizations, the Dutch Diabetes Association, and different regional care organizations. Knowledge on mHealth was not required. All interested in participation received an information letter about the project and an informed consent form. Informed consent was obtained before the data collection. All stakeholders who had the intention to work together with the researchers, as coresearchers, were included in this citizen science project.

### Coresearchers

Table 1 shows the different data sources (column 1), which include previously acquired data and new data, with number (column 2) and function of all involved stakeholders (column 3). As coresearchers, they were involved in different phases of research design, execution, and evaluation.

**Table 1:** All data sources, number and function of coresearchers, and coresearcher IDs corresponding with results.

Data source	Coresearchers, n	Function of coresearchers	Coresearcher ID
Questionnaires on desired involvement in citizen science research (previous [12])	160	Patients with T2DM^a^	—^b^
Questionnaires on knowledge and use of technology for diabetes (previous [8,9])	103	Patients with T2DM	—
Follow-up interviews on knowledge and use of technology for diabetes (previous [8,9])	16	Patients with T2DM (10 users and 6 nonusers of technology)	Patient 1-16
Interviews on expectations before using a technology for diabetes (previous [7,9])	27	Patients with T2DM (n=25) and lifestyle coaches (n=2)	Patient 17-43
Follow-up focus groups on experiences after using a technology for diabetes (previous [7,9])	25	Patients with T2DM	Patient 17-44
Recurring cocreation sessions (previous [9])	4-6	Patients with T2DM	Patient 45-50
Interviews with development companies on technology for diabetes (new)	6	Developers of 4 companies	Developer 1-6
Focus group with healthcare professionals on digital care for diabetes (new)	8	Health care professionals	Professional 1-8
Interviews with specific stakeholders on implementation of digital care for diabetes (new)	10	Employees of the Dutch Diabetes Association (n=2), health care professionals (n=3), management or overarching function (n=4), and involved in development of care guidelines (n=1)	Professional 9-18

^a^T2DM: type 2 diabetes mellitus.

^b^Not applicable.

### Previous Data Collection

The dataset of this study consists of earlier collected data and newly obtained data (Table 1). In previous research, we have performed questionnaires about patients’ preferences regarding their involvement in citizen science research [12]. Data were collected from 160 patients with T2DM. Second, questionnaires were collected from 103 patients with T2DM to investigate their knowledge on and use of technologies for diabetes [8,9]. Third, as a follow-up study to these questionnaires, semistructured in-depth interviews were conducted on the knowledge and use of technologies for diabetes [8,9]. These interviews were conducted over the internet with 16 patients with T2DM, 10 of whom were users of technology for diabetes and 6 of whom were nonusers of technology for diabetes. Fourth, 25 patients with T2DM were invited to test an mHealth for T2DM, and 2 lifestyle coaches were involved to assist some of the patients with the process [7,9]. In this study, we collaborated with 4 companies (ie, Clear.bio, mySugr, MiGuide, and Selfcare) with whom we organized a webinar to inform the patients about the mHealth apps and gave the patients the ability to choose their preferred technology. Before the testing phase started, we conducted semistructured interviews with the patients and lifestyle coaches about their expectations regarding the technology. Fifth, after the testing phase we organized focus groups in collaboration with the patients [7,9]. These focus groups aimed to discuss their experiences with and after using the technology. Finally, we organized a series of cocreation sessions to understand the desired direction of research on technologies for patients with T2DM, according to the patients themselves [9]. A group of 6 patients was formed and 4-6 of them were present at each session. At the end, the group made an agenda for follow-up research directions and wrote a letter of recommendation to the Dutch government.

### Data Collection of New Data

#### Interviews With Developers

Data were collected between October and November 2021. Semistructured in-depth interviews were conducted with 4 companies (ie, Clear.bio, mySugr, MiGuide, and Selfcare). A total of 6 developers of technology for diabetes who were employed at the 4 development companies and 1 lifestyle coach became our coresearchers. The interviews were conducted over the internet and lasted between 60 and 105 minutes. During each of the interviews, 2 researchers were present—1 had a leading role, and the other researcher took notes, and audio recordings were made. The interviews focused on the experiences of the companies in developing digital technologies specifically for patients with T2DM and an outlook toward future developments or apps within their technology. Furthermore, we discussed findings including experiences regarding the use of mHealth among patients with T2DM [7,8] and how the companies can use these findings.

#### Focus Group With Health Care Professionals

Data were collected in June 2022. In addition, 1 focus group was organized in which 8 health care professionals participated. All professionals were involved in care of patients with T2DM, both in general practice and hospital settings. The group of professionals consisted of 3 general nurse practitioners, 4 diabetes nurses, and 1 lifestyle coach. The focus group lasted for 120 minutes. Furthermore, 5 researchers were present of whom 1 took extensive notes, and an audio-recording was made. In total, 3 statements, developed in cooperation with patient coresearchers, were discussed, that are (1) the use of digital technology in care adds value to the treatment of patients with T2DM, (2) I should use more digital technologies in the treatment of patients with T2DM, and (3) I have a crucial role in the implementation of digital technologies as part of the treatment of patients with T2DM. A discussion on these statements was followed by a discussion on the needs for education and training.

#### In-Depth Interviews With Stakeholders in Health Care

Data were collected between October and November 2022. A total of 10 semistructured in-depth interviews were conducted with different stakeholders related to the care system or the care of patients with T2DM. These stakeholders included 2 employees of the Dutch Diabetes Association, 3 health care professionals, 4 professionals with a management or overarching function, and 1 stakeholder involved in the development of general practice guidelines. All interviews were conducted over the internet with 1 or 2 researchers present and lasted for 30-60 minutes. Notes were taken during the interviews and audio recordings were made. The interview guide was based on the NASSS framework [11,13], which meant that all 7 domains were included in the data collection. The interviews with health care professionals focused more on value proposition and adopters before getting into depth to the other domains, and the interviews with the stakeholders with management or overarching functions focused on the organization and wider system. All interviews ended with the domain embedding and adaptation over time.

### Data Analysis

All data, meaning the previously and newly obtained data, were combined and analyzed. Descriptive statistics were applied to the quantitative data [8,12] to analyze willingness to participate in research, preferences about methods of participation, motivation and competencies to participate, the actual use of apps, performance expectancy, effort expectancy, social influences, and facilitating conditions. Regarding the qualitative data, all audio recordings were transcribed verbatim and combined with extensive observation notes. A deductive data analysis was applied based on the domains of the NASSS framework [11]. The transcripts and observation notes were read, and codes were assigned to fitting passages. After discussion with the entire research team, 1 researcher coded the data. The findings were discussed with the research team during biweekly analysis meetings. Software package NVivo 11 (Lumivero) was used to analyze the qualitative data. Data saturation was achieved when no new themes emerged.

### Trustworthiness

We used several procedures to obtain credibility and transferability [14]. Questionnaires, interviews, and focus groups were conducted to increase the credibility by method triangulation. Investigator triangulation was reached since 6 researchers were involved in the study design, data collection, and analysis of all datasets. Furthermore, this team consisted of researchers from different research institutes, and in several research phases, patients with T2DM collaborated as coresearchers. Peer debriefing took place at weekly meetings with the research team, where both scientific and organizational aspects were discussed. Summaries with findings of different research phases were shared with the coresearchers as part of the member check, and preliminary findings were shared and discussed during a workshop at a care festival in the Twente region. A thick description was developed for transferability to a different context.

### Ethical Considerations

Ethical review and approval were obtained from the Ethics Review Committee of the University of Twente (210043). The coresearchers provided written informed consent and were informed about their right to withdraw at any time. Data were anonymized, and data confidentiality was maintained. All participants were informed about the study and their right to withdraw at any time.

## Results

### Overview

The results are categorized according to the 7 domains of the NASSS framework [11]. We aimed to explore perspectives and challenges on the implementation of mHealth in T2DM care pathways. The findings include quotes from the involved patients, health care professionals, and other stakeholders involved in health care. An overview of the main findings is presented in Table 2.

**Table 2:** The main findings for each of the 7 domains of the NASSS^a^ framework [11].

NASSS framework	Main themes	Findings by the coresearchers
Condition	Lifestyle Self-management Listen to your body Learning	You have to change your lifestyle after the diagnosis T2DM^b^. Changing your lifestyle is a learning process. Listening to your body is crucial.
Technology	Usefulness Multiplicity of apps Connecting Information provision Usability Availability	Technology could assist in changing your lifestyle. There is rapid development of new apps. It is challenging to combine and connect different apps. Information on usefulness of the technology is necessary. Unclear for whom the technology is available and what it costs.
Value proposition	For patients with T2DM: Insight Alarms Self-management Value For care professionals: Explainability Personalized care Insight Pressure on care system	For patients with T2DM: To know what happens to your body. Receiving continuous signals through an app. Increased self-management and self-reliance. Personal values are most important. For care professionals: Provide specific explanation for an individual patient. Obtain more information about the individual course of the disease. Lower pressure on health care.
Adopters	Multiplicity of apps Learning Responsibility	More clarity is needed about the most suitable apps. Training for care professionals. Unclear who will introduce the technology in practice.
Organization	Shared vision Comparison with current Work pressure Responsibility Resistance	An overview of who is responsible for what is needed. Create a shared vision and pathway for digital health care together. Digital care must be as good as or better than current care. Workload is already high in many organizations. Dealing with resistance among care professionals is difficult.
Wider system	Benefits Multiplicity of organizations Reimbursement Care guidelines Stigma	Multiple institutes are involved in decision-making regarding health care innovations. Long-term health benefits and costs are important. T2DM has to deal with its stigma. Lack of reimbursement is a crucial topic. The Dutch care guidelines are leading in care practices.
Embedding and adaptation over time	Normal future Personalized care Pressure on care system Gradual implementation	Telemonitoring and digital health will be normal in the future. Digital care will reduce pressure on the healthcare system. Care pathways will be personalized per individual.

^a^NASSS: nonadoption, abandonment, and challenges to scale-up, spread, and sustainability.

^b^T2DM: type 2 diabetes mellitus.

### Condition

T2DM is one of the most common and complex chronic conditions in the Netherlands, with an enormous impact on quality of life and health care costs. It is expected that the number of people with T2DM will only increase further in the coming years due to an aging population and an unhealthy lifestyle. Patients with T2DM shared that they had to change their lifestyle.

In the beginning it was very hard to get used to the fact that I have diabetes. I was not allowed to live my life anymore. For now, it is just part of my life.

Patient 13

A healthy lifestyle plays a fundamental role in the prevention and treatment of chronic diseases such as T2DM. It has a positive effect on glucose regulation, use of less or even no medication, and can lead to significant improvements in risk factors for complications such as cardiovascular disease.

Something changed when my doctor told me that I might need insulin. The possibility to live with less medication became a driving force to start changing my lifestyle.

Patient 6

However, adapting their lifestyle and coping with T2DM is a learning process in which patients must learn to understand and listen to their body.

Listening to my body helps in coping with the diabetes.

Patient 1

When someone is capable of understanding the development of complications, such as dangerously low blood glucose levels, they can possibly prevent it. Various lifestyle factors such as nutrition, physical activity, sleep, and stress play an important role in the lifestyle management of patients with T2DM.

### Technology

Technology has the potential to support patients with T2DM in providing better insight into their health, greater awareness of their symptoms, insight into the effect of various lifestyle factors on their condition, and the capacity to manage their own health. Among all available apps, our patient coresearchers claim that apps including tailored education, personal data analysis, personalized feedback, and the option to communicate with a care professional are most effective. The care professionals worried about the compatibility of apps with sensors, personal devices, and electronic patient records. To avoid information differences, it is necessary that information is available to patients and professionals in order to properly use the technology and understand what it is for. Another issue regarding technology was costs and thereby low inclusiveness of technology.

Innovations and apps like that are only for the happy few, that’s what it comes down to.

Patient 46

In addition, possible differences between younger and older patients are mentioned.

The younger people, yes, they have already grown up with that, they are so mature with the mobile. For them it will be easier, yes.

Professional 3

Although this is an opinion most have, it is not always the case that older patients cannot use it.

It is very special to see which people can and cannot join and that is sometimes surprising, very often surprising that someone 90 plus and then use it without any problems.

Professional 4

### Value Proposition

Based on the interviews and focus groups, we identified different kinds of values and asked 4 patient coresearchers whether they recognize these values and how they can be prioritized. Table 3 shows these values and how they were prioritized.

**Table 3:** Six values, prioritized and discussed by coresearchers.

Priority	Value	Example
1	Personal value	Happiness, convenience, and health
2	Value for health care system	Better health care and cooperation
3	Moral value	Justice, fairness, freedom, privacy, and autonomy
4	Technical value	Better technology, usability, and integration
5	Business value	Efficiency and effectiveness
6	Commercial value	Money, revenue, profit, and savings

This distinction helped to have a more nuanced discussion on the values of technology from the perspective of patient coresearchers. One of the coresearchers claimed the following:

…personal values should come first and business values should come last.

Patient 45

In addition, all reached the general consensus that personal values must come first and money-oriented values last. An important remark with the latter is that everything should be paid for and therefore money-related value cannot be ignored. While discussing how personal values can go together with the interests of, for example, health care insurance companies, a coresearcher said the following:

we should come closer together and find a way in the middle.

Patient 50

Professionals thought it was valuable that patients know what happens to their bodies during the day. Information through the app could provide a type of coaching for patients and continuous monitoring of changes in their health. In addition, mHealth apps can increase self-management and self-reliance; however, this requires a certain level of intrinsic motivation of patients.

I think that the sense of independence and self-reliance and the responsibility for how they manage the disease, that puts much more on the patient. This could also ensure an equal relationship with the healthcare provider, because they have insight and can form an opinion about it.

Professional 4

According to professionals, technology provides more specific information about what diabetes does to the body of an individual patient thereby preventing the progression of the disease. In addition, professionals stated that apps can lower the pressure on health care and save time. Although these aspects remain a point of discussion and a question mark, health gains are underlined by everyone. As 1 lifestyle coach argued:

…by using this type of technology, you can prevent a progressive course of the disease, or go into remission and use less medication during treatment. This can ultimately lead to a reduction of the pressure on the healthcare system.

Professional 11

### Adopters

In our previous research, we explored the adoption of mHealth apps among patients with T2DM. The main reason to use apps was the possibility to manage diabetes and gain insight in your personal health. However, using apps and keeping track of the personal data was time- and energy-consuming, and professionals were often not supportive or actively encouraging the use of apps [7-9,12]. Our new data provided similar findings, because there were still few professionals that used apps in their practice. To improve this, more information is needed about which apps are most suitable or user-friendly. More and more care practices provide patients the opportunity to view their files digitally and share personal health data with the care professional.

As a healthcare professional, it is very nice to see that people get to work with the information on our platform. It’s just not fun, and that has of course happened for years, when we told the same story three times a year and then that doesn’t help.

Professional 7

To use technology together with patients, training seems necessary for the health care professionals.

A point of discussion is who will explain the options and information about available apps to the patients. The health care professionals with whom they have already built a relationship of trust seem the best option. Furthermore, a solution is using apps in a collaborative way. It will have to become something of the patients and health care professionals together, especially at the start.

One-on-one attention must continue alongside the app. Especially in the beginning, later the app can take over.

Professional 3

When the professional can view the patient’s data and thus provide targeted advice, it is more likely something will be done with this advice compared with advice without the information provided by the app. One professional experienced that her patient followed advice she had given multiple times, but the advice was not followed by the patient before, but this time she provided feedback based on her personal data. The use of patient data in providing personalized feedback is experienced as pleasant and fun by professionals. Furthermore, it is more difficult for the patient to give desirable answers to questions, because the professional can actually check.

After all these years, they know what I want to hear. So, they also show desirable behaviour during consultation hours.

Professional 2

### Organization

For the organization of digital care for diabetes, it is necessary that there is a basic list with information about available and reliable apps. In addition, the path for digital health care needs to be designed together, involving patients, care professionals, and care organizations. This pathway should be integrated into the current care practices for diabetes according to a coresearcher from a regional care organization.

I think you need to have some kind of care pathway in which it is normal to use an app. The management should really stand for that, fed by their healthcare professionals to make agreements.

Professional 14

It certainly seems possible, but it is necessary to take small steps to achieve these pathways. Also, the care provided with apps must be as good as or better than current care, otherwise no transition will be made within organizations.

If you want to replace face-to-face with digital care, it must have at least the same quality and yield the same effects, otherwise you will not take that step. From that point of view, we think it’s possible, that is already positive.

Professional 12

However, current health care challenges, such as staff shortages, could enhance the transition because less staffing is required, and apps could assist with easier and faster access to care.

Some organizations do not offer digital care, and professionals only respond to it if the patient asks about apps. Not all organizations are willing to invest extra time to innovate. This is mainly due to high workload and underlines the importance of insight into the added value of innovations. We had regular conversations about resistance among health care professionals, mainly because they have to adapt their way of working.

But the resistance at an individual level, that staff should do something different from what they are used to, is enormous. If they do not participate, then the patients will not participate at all.

Professional 16

To improve the implementation of technology in diabetes care, there must be a clear vision and strategy within organizations.

A very clear vision should provide direction. And as far as I’m concerned, the step we need to take is to make concrete choices.

Professional 14

When this is in place, most health care professionals go along with it and determine the care pathway and the information they provide to patients.

### Wider System

When reflecting on society, T2DM has to deal with stigma. As our coresearchers mentioned, they often hear that T2DM is their “own fault,” whereas many external factors play a role. The entire society will have to be organized differently, such as products in supermarkets. Connected to this issue of stigma, financial reimbursements remain an issue and a difficult point on all fronts. The Dutch Government or national organizations must define the ambitions, which care organizations and practices can work on. Also, regulations are needed to properly manage aspects related to privacy and data.

If medical technology takes such a huge flight, regulations must be introduced, even though there is also a kind of medical professional secrecy, it is of course extremely lucrative if you can sell all this health data.

Developer 2

Collaboration between patients, hospitals, and other care practices is necessary to understand all needs. Furthermore, involved managers have to steer or control the processes in all instances. Besides control by management, physicians and nurses must become leaders within their organizations.

Care practices need to take their own decisions regarding the use of apps and which apps since there is no intention to develop Dutch care guidelines on apps.

We will never include something about specific apps in the care guidelines. In a manner of speaking, we will not include what can be effective or helpful, because we want to act independently and will therefore never provide a list of apps.

Professional 18

Currently, patients already use a variety of apps that are unknown by the care professionals. Not only professionals but also a patient’s social network can help in gaining knowledge about apps and also in using the apps. It is now assumed that this is only necessary for older patients; the younger patients will know and can do everything themselves.

If you think about the older patient, then knowing about apps can really be something for the social networks or the informal caregiver who sees what it’s all about or where they can support.

Professional 10

### Embedding and Adaptation Over Time

When considering the implementation over time and changes to health care settings, all health care professionals agree that where it is still an exception now, telemonitoring or digital care will be standard care in the future.

I expect many more people to use the online applications, many more patients, I believe. And I hope the healthcare providers too.

Professional 1

Digital care might even reduce the pressure on the health care system, and it might lower the costs of diabetes care. It is becoming easier and better to offer personalized care to patients, developed within a generic care path for digital care. Another way of consulting will be determined and implemented which is largely hybrid care and, in addition, consultations more on request than periodic check-ups.

If a trend goes in the wrong direction, you can signal it earlier, but also when things are going well, there is no need to contact the healthcare provider. When interaction is needed, that it becomes accessible, so that you can send an app and that you do not have to visit the doctor or the hospital to get advice.

Professional 7

Some of the professionals think that these changes in digital care and monitoring for diabetes will become the end point in diabetes treatment.

Especially when it comes to regulating glucose levels. I think app use will really be a revolution in the treatment of diabetes, once it’s through and on the market and reimbursed, 30 years from now you’re going to see people with barely any complications from diabetes. I really think this is going to be a final destination for healing.

Professional 14

## Discussion

### Principal Findings

This study aimed to explore perspectives and challenges on the implementation of mHealth in T2DM care pathways. The domain condition showed that being aware of their condition and changing lifestyle were crucial for patients with T2DM to get to grips with their life. Regarding the domain technology, there is a rapid development of mHealth apps, and users should know and understand how to use them to achieve assistance in lifestyle. The domain value proposition was divided in patients with T2DM who elaborate on personal values and care professionals who elaborate on personalized care and lower pressure on health care. For the domain adopters, it is crucial to know who needs to use and introduce the apps in practice. On the organization domain, responsibility, a shared vision within organizations, and resistance among health care professionals were mentioned as important. The domain wider system showed the importance of involvement of multiple institutes and reimbursements. However, digital and personal care will become the normal future when regarding the domain on embedding and adaptation over time.

Although care professionals of this study argue that it will become care as usual in the future, at the moment, they are not taken action to start the transition toward including apps in care pathways. The different stakeholders know about (some) technological possibilities but seem to procrastinate implementation in their care pathways. There are some early adopters, such as Santeon [15]. Santeon is the Dutch hospital group in which 7 top clinical hospitals collaborate openly with the aim of improving medical care through continuous innovation. In 2022, Santeon initiated the “Zorg bij Jou” (Care with you) program, which aims ultimately to nationally implement uniform hybrid care pathways with digital services, such as telemonitoring and internet-based consultations. These services are centrally coordinated from a medical service center. Another goal is to implement this program for more than 30 (chronic) health conditions within 5 years to lower health care consumption and costs against the same or higher quality of care, and to facilitate more integrated and tailored care. In this way, a national open platform is being built with 24×7 services, where other health care services can also make use of, making it scalable to other types of health care and social services as well. These kinds of early adopters aim to get more health care professionals to join and implement hybrid care. Some coresearchers argued that they want to incorporate the technology as part of care, if it was tested by several others, for example, general practitioners. Our coresearchers claimed that a group of committed innovators, including patients, care professionals, and management stakeholders, seems needed to reach a specified ambition and prove the effectiveness of apps. However, among current care professionals, it seems hard to find this small group of innovators. Furthermore, the involved professionals point to a lack of an overarching vision by organizations. This vision could also assist in lowering the fragmented implementation approach in care practices. Often the organizations take a top-down approach and only involve care professionals and patients in a later stage [16,17]. With our citizen science approach, we show the desire from all different stakeholders to be involved in the development of a strategy toward the best implementation of apps in the care pathways.

The health care guidelines shape care pathways on the basis of evidence-based medicine. Evidence-based medicine remains needed to provide care for which a professional could take responsibility [18]. However, current ideas about personalized medicine slightly takes the focus off evidence-based medicine [19] and give professionals the opportunity to share responsibility with their patients. A professional often decides whether a technology is suitable for the patient or not. However, there is gradual change from paternalistic to shared decision-making [20,21]. In shared decision-making there is a need for the professional to actively assist the patients in preference elicitation and align these with the patients’ unique situation and preferences for care [12]. Introducing and discussing a technology could not only become part of shared decision-making but also share responsibility.

The determinants acquired from our study can form a base toward strategies. To improve the uptake of mHealth by all different stakeholders, strategies that complement each other seem necessary. First, there is a need for commitment of organizations to use the knowledge and experience of patients and health care professionals with regard to the available range and proven effective apps. Second, there is a need for commitment of patients with T2DM to use apps, and of health care professionals to adapt their work process where technology is proven as a self-evident solution direction. Third, the implementation and adaptation of care and work practices needs to be gradual with assistance from management or more overarching organizations with a clear ambition for future care provision. And fourth, a learning network, in which the added value and effectiveness of technology are regularly examined together, could be created taking the user and their context into account.

### Strengths and Limitations

To get a more extensive understanding of different perspectives, we used a citizen science approach with a multiplicity of stakeholders. Collaboration during this study offered added value, because coresearchers have firsthand experiences with the disease, the use of apps, care practices, care professionals, and health insurance. The insight of coresearchers enriched our understanding and led to a wider perspective. Involving coresearchers can be seen as a moral obligation for researchers but has several benefits. Being a coresearcher can have direct mental and physical health benefits since it actively involves people with their own health, brings purpose, a social network, and possibly even acceptance of their condition. It thereby has several benefits for the research itself. Coresearchers often have better access to other citizens and know important issues from experience and therefore enhance the relevance of the research. Furthermore, the coresearchers can guide the researchers toward the most relevant directs of current and future research by sharing their experiences. Using the NASSS framework strengthens both the researchers and coresearchers to dive into not only the value for the patients, but also their vision toward societal embedding.

As Schoville and Titler [22] argue, most models on adoption focus on the end user of the technology and implementation models mainly consider the necessary changes and methods of interventions. We applied the NASSS framework because it offers a complete approach to study the multiplicity of health care stakeholders and the variety of perspectives on the implementation of mHealth. Although most of the involved coresearchers are already interested in T2DM innovation, the involvement of this multiplicity of stakeholders is a main strength of this research. The different domains of the NASSS framework complement each other, and in each of the domains a different multiplicity of stakeholders could be incorporated. The NASSS framework is a combination of different implementation models that complemented each other into this framework consisting of 7 overarching domains. In comparison to other implementation models, the NASSS framework focuses more specifically on the current state and the future, besides the actual implementation process. The generic implementation framework (GIF) seems to have most resemblance with the NASSS framework [23]. Both are designed to take a variety of perspectives into account when designing an implementation effort. Differences between these frameworks are the additional focus on future embedding in the NASSS framework, and where the concepts within the GIF are seen as a memory aid to develop an implementation protocol, NASSS provides hands-on guidelines for the usage of each domain. As part of the NASSS framework, we discussed the embedding and adaptation over time. This domain was most difficult for the stakeholders to elaborate on. Possibly research methods that enhance creativity and reflecting on the future, could add another layer to the findings.

### Recommendations

According to our coresearchers, it is necessary to start forming an overarching vision on a regional level. A start with regional initiatives to incorporate technology in care pathways has been made during this study, which will continue. Many regional organizations already cooperate and know each other. However, on this level, it is also needed to have a similar view toward the future and needs for innovation and implementation. If there is no mutual vision on the regional level, it seems hard to continue toward national or international levels. Collaboration of researchers with all the different stakeholders on a regional level could improve the cooperation and vision within a region and afterward, translate the regional findings toward national or international levels, or perform research on a larger scale. Furthermore, regarding the practical use of the findings, our research could enhance awareness of technologies suitable for patients with T2DM, which could become part of care pathways. Based on barriers and drivers of the use and implementation of these technologies, organizations can reflect and define a strategy suitable for their organization. Also, the findings can make organizations and management aware of mHealth readiness, with a reflection on necessary changes before use and implementation are possible in view of the involved stakeholders and care process.

With regard to organizing the cocreation sessions, it was paramount to invest in close contact with coresearchers and to maintain this contact. According to the coresearchers it was best to have short meetings at short intervals. In our experience, it was relatively easy to find older male coresearchers since this group is highly motivated through their negative experiences with health care professionals and interest in technology. This group of retired men also has relatively much spare time to invest. In order to have a more heterogeneous group of coresearchers, consisting of younger people, more time needs to be spent on recruitment before starting the group. Once a group is established it can be hard for new coresearchers to become part of the group and feel safe to share personal ideas and experiences.

Most research in which the NASSS framework was applied [24,25], considered the implementation of 1 specific technology or app. This is similar for other implementation frameworks, such as the GIF, which as well focus on implementation processes of 1 specific technology [23]. Our study used the NASSS framework to gain insight in the implementation of digitalized care with a variety of apps, which is probably more difficult by applying other frameworks of implementation due to the focus on a technology and less on the context. Although the focus on 1 specific technology might provide in-depth information about the implementation of this technology, the focus on a spectrum of similar technologies with comparable aims is valuable as well. In addition to specific insight in the technology, the focus on similar technologies provides a wider understanding of the organizational structures and the system in which it might be implemented. The guidelines of the NASSS framework, and specifically the toolkit with specific questions as part of each domain [13], were useful in practice to create meaningful conversations with all stakeholders and understand the wider context. In a context in which there is a variety of similar technologies, such as T2DM, we would recommend broadening the focus when applying the NASSS framework.

### Conclusion

This study emphasized the added value to discuss the implementation of mHealth apps with different stakeholders. They attributed to the possibilities and limitations of the implementation of apps in diabetes care pathways. A clear vision for an organizational perspective and specific prerequisites for implementation is crucial to developing responsible implementation strategies at micro and meso levels. At the national level, guidelines for regulations, privacy, and security are essential, as well as the integration of mHealth into T2DM care guidelines and sufficient reimbursement by health insurers. The context has to change to ensure that mHealth becomes accessible to all patients with T2DM, regardless of personal financial capabilities or the severity of the disease, and to shift the focus toward T2DM prevention using apps as support.

The following were this study’s contributions to literature:

1. Building on literature on patients and health care professionals, this study provides a complete overview of opportunities and challenges expressed by multiple stakeholders in care of T2DM.
2. Previous studies applied the NASSS framework to investigate a specific digital health app. This study shows that the NASSS framework could also provide insight into the implementation of digitalized care with a variety of apps.
3. The use of citizen science methods in this study follows and contributes empirical examples to the existing literature on citizen science.

## Abbreviations

GIF: generic implementation framework
mHealth: mobile health
NASSS: nonadoption, abandonment, and challenges to scale-up, spread, and sustainability
T2DM: type 2 diabetes mellitus

## Data Availability

All relevant data are in the manuscript and its supporting information files. The dataset that supports the findings and conclusion of this study is available from the corresponding author on reasonable request. The data are not publicly available due to privacy and/or ethical restrictions.
